# Hatha yoga, integrating the segmental stabilization exercise model, can improve trunk muscle endurance in healthy adults

**DOI:** 10.3389/fpubh.2024.1487702

**Published:** 2024-11-25

**Authors:** Maja Petrič, Lijana Zaletel-Kragelj, Miroljub Jakovljević, Renata Vauhnik

**Affiliations:** ^1^Faculty of Health Sciences, Department of Physiotherapy, University of Ljubljana, Ljubljana, Slovenia; ^2^Faculty of Medicine, Department of Public Health, University of Ljubljana, Ljubljana, Slovenia

**Keywords:** spine stability, prevention, physical exercise, low back pain, yoga, muscular endurance

## Abstract

**Introduction:**

Low back pain is a very common symptom worldwide and an increasingly important public health problem. Exercises to stabilize the lumbar spine and pelvis have been shown to be effective in preventing or reducing the risk of low back pain. Hatha yoga practice is now increasingly appearing in prevention research and has the potential to improve trunk muscle endurance. The prevention research on endurance training of trunk muscle lacks evidence. The aim of this study was to investigate the effectiveness of a professional and scientifically based exercise program to improve and balance trunk muscle endurance in healthy adults.

**Methods:**

A non-randomized control study was conducted. Participants were healthy adults without low back pain or injury who had not performed exercises to improve trunk muscle endurance prior to participation in the study. An analysis of the proposed exercise program’s effectiveness was conducted. The participants were divided into two groups: the exercise group (EG) performed a three-month hatha yoga exercise program (60 min twice a week) that included the spinal and pelvic segmental stabilization exercise model, or the control group (CG), in which participants were asked to maintain their previous lifestyle during the study period. Trunk muscle endurance was measured at baseline (PRE) and after completion of the exercise program (POST) in both groups. The changes in the measured variables were analyzed (PRE-POST analysis, EG-CG comparison).

**Results:**

Seventy-two subjects (n_EG_ = 36, age 32.2 ± 6.8 years; n_CG_ = 36, age 29.9 ± 7.8 years) participated in the study. After the exercise program, the EG participants had significantly better results in endurance in three of the four trunk muscle groups (*p* < 0.05), but not in any of the endurance ratios.

**Conclusion:**

Hatha yoga when integrating the segmental stabilization exercise model can significantly improve the endurance of at least three of the four major trunk muscle groups. For clinical relevance, the long-term effects of the proposed exercise program should be investigated in individuals with low back pain.

## 1 Introduction

Low back pain (LBP) is a very common symptom worldwide and an increasingly important global public health problem (1). In 2019, LBP was the leading cause of functional disability (2), and retained its leading position even after the emergence of COVID-19 pandemic (1). It occurs in all age groups, with prevalence, incidence and years lived with disability higher in women than in men, but increasing with age in both genders (1–3). It is estimated that LBP reoccurs in about 33% of cases within 1 year of the first episode and increasingly becomes a chronic condition (3, 4). Some lifestyle factors (such as smoking, obesity, insufficient physical activity and sedentary lifestyle) are associated with the occurrence of LBP and the further development of musculoskeletal problems (3, 5, 6).

Adequate muscular endurance of the back and abdominal muscles is an essential component of low back stability and injury prevention (7, 8). Therefore, inadequate or inappropriate trunk muscle performance or an inappropriate ratio of muscle endurance between the major trunk muscle groups (extensors, flexors and the lateral trunk muscles) are important risk factors for the occurrence of LBP (7, 9, 10).

Researchers are mainly investigating approaches to alleviate pain intensity and improve functional abilities in patients with (chronic) LBP (11). On the other hand, there is a lack of studies investigating effective interventions to prevent LBP and reduce the recurrence of LBP (11, 12). One of the known evidence-based effective interventions to prevent LBP is exercise—alone or in combination with LBP prevention education (12, 13). Lumbar spine stabilization exercise programs based on the segmental stabilization exercise model (14) have been shown to be effective in improving trunk muscle endurance and reducing the recurrence of LBP (15, 16). The segmental stabilization exercise model incorporates the principles of motor learning theory and refers to the re-establishing of simultaneous contraction of the deep trunk muscles (transversus abdominis, pelvic floor muscles, diaphragm and deep multifidus) and major superficial trunk muscle groups (14). The progression in this exercise model consists of three stages of segmental control: (1) local segmental control training, (2) closed chain segmental control training, and (3) open chain segmental control training (14).

In recent years, yoga has been increasingly studied as one of the effective treatment strategies for LBP (17). Most of the available studies in the field of yoga research investigated the efficacy and appropriateness of yoga as a therapeutic approach in (chronic) LBP patients, less is known about its preventive outcomes (18, 19). Yoga is a mind and body practice whose name comes from the Sanskrit root “yuj” meaning ‘union’, ‘to unite or to connect’, ‘to integrate’ (20). There are four traditional types of yoga however, hatha yoga emphasizes on the importance of physical fitness more than the other three (20). Through various techniques, hatha yoga strives for a dynamic balance between strength and flexibility that takes place on a physical, mental and emotional level (20, 21). A regular hatha yoga practice can improve the endurance of the trunk muscles and the low back stability (22–29).

The static holding of asanas is one of the basic principles of hatha yoga, which gives this type of practice the potential to strengthen the trunk muscles. The practitioner must be able to maintain trunk stability and thus a neutral position of the lumbosacral spine, even when the asanas increase in difficulty (e.g., by isolating movements of the upper and/or lower limbs while maintaining static trunk stability) (30, 31). Similar to the segmental stabilization exercise model, the complexity of techniques in hatha yoga is gradually increased and improved. For example: performing asanas in both open and closed kinetic chains; performing different breathing techniques to gradually calm the breath and increase the length of inhalation and exhalation; increasing the time spent performing or holding each asana or exercise; etc. (32).

As mentioned above, there are several studies on the effectiveness of yoga in improving trunk muscles, albeit with very heterogeneous measurement methods and interventions (33). Furthermore, to our knowledge, there is no study in which authors have investigated the effect of a hatha yoga exercise program that incorporates the principles of the segmental stabilization exercise model into this exercise program.

Aiming to provide evidence for an effective public health intervention to prevent LBP or reduce the risk of recurrence of LBP, the objective of this study was to investigate the effectiveness of a professional and scientifically based hatha yoga exercise program that integrates the principles of the segmental stabilization exercise model to improve and balance trunk muscle endurance in healthy adults.

## 2 Materials and methods

### 2.1 Study design, time frame and setting

This research was designed as an interventional study. It was conducted from September 2019 to March 2022, at the Faculty of Health Sciences at the University of Ljubljana.

### 2.2 Target population and sampling

Potential candidates were invited to participate in the study via electronic media (email, Facebook, etc.) and chain reference sampling. The following inclusion criteria were considered for participation in the study: (1) healthy adults between the ages of 20 and 45 years, (2) without LBP at the time of enrollment in the study, (3) without musculoskeletal injuries or other diseases that could be a contraindication to the muscle endurance test or pose a risk to the individual’s health, and (4) who did not perform yoga practice (continuously, at least once a week) or exercise programs to stabilize the lumbar spine. The aim was to obtain at least 35 participants in each group, and in view of possible dropout, several participants were intentionally included in the study. All participants who met the inclusion criteria signed an informed consent form upon entry into the study.

Participants were assigned to one of two study groups: (a) an exercise group (EG) or (b) a control group (CG) according to the case–control matching method (34), taking into account gender, age and physical activity. Randomization of the subjects into EG and CG groups was not possible for implementation reasons, as sufficient motivation and time of the participants—especially in the EG group to participate in the regular training sessions—was crucial for the implementation of the study. Potential candidates who met the inclusion criteria were given the opportunity to choose a group (EG or CG).

### 2.3 The study course

#### 2.3.1 Exercise program

The EG participants completed a three-month hatha yoga exercise program that integrated the principles of the segmental stabilization exercise model. The exercise program comprised a total of 25 training sessions, which were performed twice a week for 60 min each. Each training session consisted of asanas (yoga postures) and pranayamas (controlled breathing techniques), starting with gradual warm-up exercises (10 min), followed by the main part of the training session to strengthen trunk muscle endurance (35 min) and gradual stretching and relaxation exercises at the end of the training session (15 min). The asanas were gradually intensified during the exercise program, e.g., through more repetitions (dynamic performance of the exercise), holding the position for longer (static performance of the exercise), linking the asanas into sequences, simultaneously performing pranayama during the asanas or asana sequences, etc. In accordance with the segmental stabilization exercise model, the simultaneous contraction of the deep trunk muscles in each asana was emphasized and different positions or dynamic movements of the upper/lower limbs (in closed or open chain) while holding the basic asana were gradually performed during the exercise program.

The exercise program consisted of low to moderate intensity exercises. As the low to moderate intensity exercises are described by ratings of 9–15 (35, 36) on the Borg Rating of Perceived Exertion (RPE) scale 6–20 (37, 38), the exercise program was graded within these ratings as rated by the participants for each training session. To maintain the quality of the therapeutic approach, the three-month exercise program was conducted with a maximum of 11 participants per each training session. The exercise program was led by a physiotherapist with a master’s degree, who is also a yoga teacher (YT 500), with several years of experience in both professional fields - physiotherapy and teaching yoga. Due to the lockdown caused by the COVID-19 pandemic, some of the training sessions in the second half of the exercise program had to be conducted online (max. six training sessions), with both the yoga teacher and the participants having their cameras switched on to ensure the quality of the therapeutic approach.

#### 2.3.2 Control group

The CG participants were asked to maintain their current lifestyle and level of physical activity during the study period and to change as little as possible. After completion of the study, CG participants were also offered participation in the exercise program.

### 2.4 Study instruments

First, the presence of contraindications and exclusion criteria was checked using a questionnaire on the demographic data, health status and physical activity of the participants (39). Before the measurements were taken, basic demographic information about the participants was obtained, as well as information on selected lifestyle characteristics, namely the amount of physical activity in a typical week, the amount of sitting in a typical day and previous experience of LBP. Body height and mass were then measured, from which the body mass index was then calculated for each participant.

The endurance of the main trunk muscle groups was then measured using four test positions, all of which were performed on a 45° Roman chair: the trunk extensor isometric hold test (EX test), the trunk flexor isometric hold test (FL test) and the trunk lateral isometric hold test for the left (L-LM test) and right side (R-LM test). The test positions, procedures and calculations of the trunk muscle endurance ratio were performed according to the protocols previously described in details in Petrič et al. (40). The endurance of the trunk muscles was measured for the first time when the participants were included in the study (PRE) and for the second time after completion of the exercise program (or after the three-month study period without training for CG participants; POST).

### 2.5 Methods of analysis

After checking the normal distribution of the variables, the measurements at the beginning of the exercise program (“PRE” measurements) and the measurements at the end of the exercise program (“POST” measurements) were statistically compared using the t-test for related-samples or non-parametric Wilcoxon signed test for related-samples. Changes in trunk muscle endurance were also compared between EG and CG (t-test for unrelated-samples or Mann–Whitney U-test for unrelated-samples).

Statistical significance was set at *p* ≤ 0.05 for all analyzes. Data analysis was performed using IBM SPSS Statistics 26 (IBM, New York, United States) and an Excel program (Microsoft Corporation, Washington, United States).

## 3 Results

### 3.1 Participants

Eighty-five participants met all inclusion criteria and 72 participants completed the study. Only those who discontinued the study of their own accord were excluded from the study. Thirteen participants dropped out of the study for various reasons (e.g., significant change in lifestyle and/or level of physical activity, pregnancy, illness, etc.). Finally, 36 participants were included in each group. There were no significant differences in baseline data between EG and CG (Table 1).

**Table 1:** Demographic data of participants for EG and CG.

	Mean ± SD or n (%)	Mean ± SD or n (%)	
	EG (*n* = 36)	CG (*n* = 36)	*p*
Gender (M/F)	n_M_ = 7 (19.4%) n_F_ = 29 (80.6%)	n_M_ = 8 (22.2%) n_F_ = 28 (77.8%)	0.772
Age (years)	32.2 ± 6.8	29.9 ± 7.8	0.187
Body height (m)	1.7 ± 0.1	1.7 ± 0.1	0.680
Body mass (kg)	65.9 ± 9.7	67.2 ± 13.2	0.662
BMI (kg/m^2^)	23.2 ± 3.2	23.4 ± 3.6	0.866

SD, standard deviation; n, number of participants; EG, exercise group; CG, control group; p, asymptotic sig. (2-sided test); M, male; F, female; BMI, body mass index.

The average participation rate in the training sessions was 85.1%, i.e., 21.3 out of a total of 25 sessions, with an average rate of perceived exertion of 10.1 ± 0.8.

### 3.2 Effectiveness of the exercise program

At baseline (PRE measurements), there were no significant differences between EG and CG in the endurance holding time (*p* > 0.05) or the trunk muscle endurance ratios (*p* > 0.05).

#### 3.2.1 Endurance holding time

After the exercise program, EG participants significantly improved their endurance in all four trunk muscle groups (*p* < 0.05), while the changes in CG were not significant (Table 2).

**Table 2:** Results of the holding time (in seconds) of the trunk muscles (comparison of PRE-POST measurements).

Group	Test	Measurement	x̄	95% CI	SD	Me	Min.	Max.	*p*
EG (*n* = 36)	EX	PRE	296.1	251.8, 340.4	130.9	285.5	99.0	661.0	**0.003** ^a*^
		POST	345.0	284.1, 405.8	179.9	286.0	116.0	1070.0	
	FL	PRE	173.0	138.3, 207.7	102.5	150.0	39.0	420.0	**< 0.001** ^a*^
		POST	263.8	208.3, 319.3	164.1	255.0	42.0	712.0	
	L-LM	PRE	129.3	117.3, 141.4	35.7	136.0	68.0	218.0	**0.014** ^b*^
		POST	148.5	129.2, 167.7	56.9	146.0	49.0	326.0	
	R-LM	PRE	120.9	108.2, 133.5	37.5	116.5	57.0	217.0	**0.012** ^b*^
		POST	142.3	123.2, 161.4	56.5	130.5	54.0	299.0	
CG (*n* = 36)	EX	PRE	362.8	266.6, 458.9	284.3	279.5	128.0	1468.0	0.888^a^
		POST	353.1	257.7, 448.5	281.9	281.5	101.0	1650.0	
	FL	PRE	194.8	147.3, 242.2	140.3	139.5	36.0	604.0	0.414^a^
		POST	208.3	159.6, 257.0	144.0	147.0	45.0	597.0	
	L-LM	PRE	135.6	114.8, 156.3	61.3	129.5	46.0	315.0	0.863^b^
		POST	136.5	117.2, 155.8	56.9	123.5	43.0	268.0	
	R-LM	PRE	118.2	99.9, 136.5	54.1	106.5	46.0	264.0	0.974^a^
		POST	117.3	99.8, 134.8	51.7	107.0	42.0	237.0	

EG, exercise group; n, number of participants; CG, control group; EX, endurance of trunk extensors; FL, endurance of trunk flexors; L-LM, endurance of lateral trunk muscles (left side); R-LM, endurance of lateral trunk muscles (right side); PRE, measurements before the exercise program; POST, measurements after the exercise program; x̄, mean; 95% CI, 95% confidence interval; SD, standard deviation; Me, median; Min, minimum value; Max, maximum value; p, asymptotic sig. (2-sided test); a, Wilcoxon signed ranks test; b, paired samples *t*-test; *The bold values are those that were statistically significant.

Significant differences were found between the EG and CG over time in terms of endurance holding time in three of four trunk muscle groups (Table 3). The calculated Cohen’s d coefficients indicate a medium and large effect (Table 3).

**Table 3:** Results of the PRE-POST differences in endurance holding time (in seconds) of the trunk muscles (comparison of the EG-CG measurements).

Test	Group	x̄	SD	%	*p*	*Cohen’s d*
EX	EG	48.9	119.7	16.5	**0.033** ^a*^	0.31
	CG	−9.6	125.5	−2.6		
FL	EG	90.8	130.9	52.5	**0.006** ^b*^	1.15
	CG	13.6	91.1	7.0		
L-LM	EG	19.1	43.7	14.8	0.086^a^	0.63
	CG	0.9	31.6	0.7		
R-LM	EG	21.4	47.7	17.7	**0.023** ^b*^	0.67
	CG	−0.9	30.5	−0.7		

EX, endurance of trunk extensors; FL, endurance of trunk flexors; L-LM, endurance of lateral trunk muscles (left side); R-LM, endurance of lateral trunk muscles (right side); EG, exercise group (*n* = 36); CG, control group (*n* = 36); x̄, mean PRE-POST difference; SD, standard deviation; %, percentage of PRE-POST improvement/difference; p, asymptotic sig. (2-sided test); ^a^, Mann–Whitney U-test for unrelated-samples; ^b^, t-test for unrelated-samples; *The bold values are those that were statistically significant; Cohen’s d, Cohen’s d coefficient.

#### 3.2.2 Trunk muscle endurance ratios

After the exercise program, only the flexor-extensor ratio improved significantly in the EG participants (*p* = 0.005), while the changes in all other ratios were not significant (all *p* > 0.363). The changes in CG were also not significant (Table 4).

**Table 4:** Results of the ratios of trunk muscle endurance (comparison of PRE-POST measurements).

Group	Ratio	Measurement	x̄	95% CI	SD	Me	Min.	Max.	*p*
EG (*n* = 36)	FL:EX	PRE	0.69	0.50, 0.88	0.56	0.58	0.10	2.87	**0.005** ^a*^
		POST	0.92	0.71, 1.14	0.64	0.79	0.07	2.92	
	R-LM:L-LM	PRE	0.96	0.87, 1.04	0.25	0.88	0.65	1.55	0.363^a^
		POST	0.99	0.90, 1.07	0.26	0.92	0.67	1.80	
	L-LM:EX	PRE	0.49	0.43, 0.56	0.19	0.45	0.20	1.03	0.878^a^
		POST	0.49	0.42, 0.56	0.21	0.45	0.14	1.05	
	R-LM:EX	PRE	0.47	0.40, 0.53	0.20	0.45	0.13	0.94	0.797^a^
		POST	0.47	0.39, 0.55	0.23	0.42	0.17	1.17	
CG (*n* = 36)	FL:EX	PRE	0.69	0.51, 0.87	0.54	0.52	0.07	2.63	0.245^a^
		POST	0.74	0.56, 0.91	0.51	0.57	0.09	2.18	
	R-LM:L-LM	PRE	0.91	0.82, 0.99	0.25	0.87	0.48	1.40	0.411^b^
		POST	0.88	0.81, 0.95	0.21	0.90	0.44	1.35	
	L-LM:EX	PRE	0.46	0.39, 0.53	0.20	0.41	0.11	0.86	0.245^a^
		POST	0.48	0.41, 0.55	0.22	0.41	0.12	0.91	
	R-LM:EX	PRE	0.40	0.34, 0.46	0.17	0.40	0.08	0.80	0.677^b^
		POST	0.41	0.35, 0.46	0.16	0.39	0.05	0.74	

EG, exercise group; n, number of participants; CG, control group; FL, endurance of trunk flexors; EX, endurance of trunk extensors; R-LM, endurance of lateral trunk muscles (right side); L-LM, endurance of lateral trunk muscles (left side); PRE, measurements before the exercise program; POST, measurements after the exercise program; x̄, mean; 95% CI, 95% confidence interval; SD, standard deviation; Me, median; Min, minimum value; Max, maximum value; p, asymptotic sig. (2-sided test); a, Wilcoxon signed ranks test; b, paired samples t-test; *The bold values are those that were statistically significant.

No significant differences were found between the EG and CG in terms of any ratio of trunk muscle endurance (Table 5).

**Table 5:** Results of the ratio of trunk muscle endurance (comparison of EG-CG measurements).

Ratio	Measurement	Group	x̄	SD	SEM	*p*
FL:EX	PRE	EG	0.69	0.56	0.09	0.879^a^
		CG	0.69	0.54	0.09	
	POST	EG	0.93	0.64	0.11	0.230^a^
		CG	0.74	0.51	0.08	
R-LM:L-LM	PRE	EG	0.96	0.25	0.04	0.543^a^
		CG	0.91	0.25	0.04	
	POST	EG	0.99	0.26	0.04	0.191^a^
		CG	0.88	0.21	0.04	
L-LM:EX	PRE	EG	0.49	0.19	0.03	0.539^a^
		CG	0.46	0.20	0.03	
	POST	EG	0.49	0.21	0.04	0.817^a^
		CG	0.48	0.22	0.04	
R-LM:EX	PRE	EG	0.47	0.20	0.03	0.140^b^
		CG	0.40	0.17	0.03	
	POST	EG	0.47	0.23	0.04	0.424^a^
		CG	0.41	0.16	0.03	

FL, endurance of trunk flexors; EX, endurance of trunk extensors; R-LM, endurance of lateral trunk muscles (right side); L-LM, endurance of lateral trunk muscles (left side); PRE, measurements before the exercise program; POST, measurements after the exercise program; EG, exercise group (*n* = 36); CG, control group (*n* = 36); x̄, mean; SD, standard deviation; SEM, standard error mean; p, asymptotic sig. (2-sided test); ^a^, Mann–Whitney U-test for unrelated-samples; ^b^, t-test for unrelated-samples.

## 4 Discussion

An exercise program that combines traditional hatha yoga with the principles of the spinal and pelvic segmental stabilization exercise model examined in our study could be an effective approach to improving trunk muscle endurance. After the three-month exercise program, EG participants achieved clinically significant improvements in endurance of all four major trunk muscle groups (mean improvement of +25.4%) compared to CG participants (mean improvement of +1.1%). The improvements in endurance in three of the four trunk muscle groups were also statistically significantly better (*p* < 0.05) than in the CG participants.

Participants rated the perceived exertion of the training sessions of the three-month exercise program as 10.1 ± 0.8 on the Borg RPE scale, suggesting that hatha yoga, which is graded according to the principles of segmental stabilization of the spine and pelvis, is a low- or moderate-intensity exercise (35, 36).

As normative values are not yet available for the trunk muscle endurance tests used in our study, we can only compare our results with the results of studies in which the authors used the same trunk muscle endurance tests. All participants in our study already showed a longer mean holding time in the LM test at the time of inclusion in our study compared to the results reported by Pagé and Descarreaux (41) (mean holding time in their study: 96.7 ± 24.9 s and 97.2 ± 21.5 s for L-LM and R-LM, respectively). Compared to the results of Ledoux et al. (42) (mean holding time in their study: 127.32 ± 74.29 s in LM test and 221.61 ± 108.6 s in EX test), the participants in our study achieved a similar mean holding time in the LM test and a longer mean holding time in the EX test at the beginning of our exercise program. It is important to note that the participants in their study were older (67.3 ± 5.1 years). No study comparing the results of the FL test could be found in the available literature.

When examining the correlation between trunk extensor endurance and history of LBP in young adults, Shaw et al. (43) found significantly poorer trunk extensor endurance after multiple repetitions of the Biering-Sørensen test in adults with a history of LBP than in those without a history of LBP. The authors found that more than one repetition of the test is required to detect impaired paraspinal and hip extensor endurance in active young adults with a history of LBP (42). Ledoux et al. (42) also found poorer trunk extensor endurance (tested with the EX test used in our study) in participants with chronic LBP compared to healthy participants.

The study conducted by Mistry (25) also measured isometric endurance of the trunk muscles (according to the protocols established by McGill et al. (9)) before and after the (hatha) yoga exercise program, and he reported some greater mean improvements in trunk muscle endurance compared to our study (improvement in endurance of extensors, flexors and the lateral trunk muscles of left and right side in their study: 33.8, 24.0, 32.8, and 20.6%, respectively). However, their exercise program was shorter (7 weeks), but the number of younger participants in his study (mean age 21.5 ± 1.5 years) was lower (*n* = 8) and, as previously mentioned, different test positions were used compared to our study (25). Similarly, in the study by Larivière et al. (44), adults with LBP (mean age 43 ± 12 years) achieved a significant improvement (*p* < 0.001) in endurance in extensors, flexors and the lateral trunk muscles after eight-week individualized lumbar stabilization exercise program as tested by protocols established by McGill et al. (9).

In addition to the better holding time of the test postures, the ratio of trunk muscle endurance is also very important for the stability of the spine and pelvis (10). Although there was no statistically significant difference between EG and CG in terms of PRE-POST changes in trunk muscle endurance ratios, EG participants improved one of the four trunk muscle endurance ratios (Table 4). By significant improvement in trunk flexors and trunk extensors endurance the endurance of those two muscle groups were much more equal and their ratio (FL:EX) was significantly better at the end of the exercise program. In EG participants also the ratio of right to left lateral trunk muscle groups (R-LM:L-LM) was closer to the optimal ratio of 1 on average at the second measurement (Table 4). In the CG participants, this ratio worsened slightly, but in neither group was the described change in the R-LM:L-LM ratio statistically significant. McGill et al. (10) describe that for good lumbosacral stability, the endurance of the right and left lateral trunk muscle groups should not differ by more than 5%. Some authors (41) have suggested in their studies that this threshold may be too strict in terms of the clinical importance of determining individual risk for LBP.

The study has some limitations. Among the most important limitations is the chosen research design. For implementation reasons, randomization of subjects into EG and CG groups was not possible, as sufficient motivation and time of the participants—especially in the EG group to participate in the regular training sessions—was crucial for the conduct of the study. As already emphasized, all potential candidates who met the inclusion criteria were given the opportunity to choose a group (EG or CG), and CG participants were also offered the opportunity to participate in an exercise program after completing their participation in the CG. Another limitation was the small number of participants. This is also due to practical limitations, because in order to maintain the quality of the (therapeutic) exercise approach, participation in each training session of the exercise program was limited to a maximum group of 11 participants. As a result, the three-month exercise program in the study was carried out in four repetitions. Final limitation could be the gender imbalance among the participants. Most of the participants in our study were women, while men made up only one-fifth of the participants in each group. This could also be due to societal attitudes, as yoga is still seen as a gentler, less intense and somehow more “feminine” type of exercise in the Western world, so men may be less likely to choose it (45). Since the exercise program in our study also incorporated the principles of the segmental stabilization exercise model, a more targeted approach to inviting the male population to practice this type of exercise (“yoga as a therapeutic exercise”) might make it more accessible to them as well (45). Furthermore, since the correction for multiple comparisons was not carried out, this could also influence our results.

In addition to its limitations, the study also has important strengths. It is the first study to examine the effects of a hatha yoga exercise program graded according to the principles of the segmental stabilization exercise model on trunk muscle endurance (both in terms of holding time and the ratio of endurance between major trunk muscles). It is also the first study to test trunk muscle endurance on a 45° Roman chair to determine the effects of exercise on the endurance of all four major trunk muscle groups.

The study has some important implications for physiotherapy and public health. Regarding the effects of yoga on the physical body, more is known about its effectiveness in improving body flexibility, but less has been studied about its effectiveness in strengthening and balancing the endurance ratios of the trunk muscles. The present study is a step toward a better understanding of the effects of yoga and its gradual introduction as a preventive or complementary form of therapeutic activity also in public health systems at the primary and secondary healthcare levels, which has already been reported as a successful approach in some countries (e. g. in United Kingdom and Portugal) (46, 47). It appears that yoga could be an important approach for maintaining physical fitness also in seniors (48). It could also be extremely useful in professions where the lower back is more stressed during work, e.g., in health professions such as physiotherapists or occupational therapists (49).

Research in this area needs to be continued. In future studies, it would be useful to include a larger group of participants and a more even gender distribution. According to the results of the statistical power analysis of the study, at least 60 participants should be included in the EG to validate the results obtained and to determine the effects of the exercise program on trunk muscle endurance. The inclusion of a larger number of subjects would be feasible if more investigators (physiotherapists and/or yoga teachers) were involved in the management of the training sessions and in the measurements. After the exercise period of the study, it would be worthwhile to follow the effects of the exercise program in the long term to observe the incidence of LBP in EG and CG participants and any changes in lifestyle characteristics compared to before the study (e.g., whether participation in the study encouraged participants to adopt a more active lifestyle). Clinical relevance should be investigated in symptomatic patients with LBP to increase muscle endurance and prevent the recurrence of LBP, as an intention-to-treat analysis should also be performed in such a clinical trial (50). If the effects of the proposed exercise program are to be further investigated in specific patient groups (e.g., those with pre-existing LBP), it would also be useful to perform functional tests.

## 5 Conclusion

A hatha yoga exercise program that incorporates the principles of the segmental stabilization exercise model can significantly improve the endurance of at least three of the four major trunk muscle groups.

## Data Availability

The raw data supporting the conclusions of this article will be made available by the authors, without undue reservation.
